# Revealing Changes in Brain Functional Networks Caused by Focused-Attention Meditation Using Tucker3 Clustering

**DOI:** 10.3389/fnhum.2019.00473

**Published:** 2020-01-22

**Authors:** Takuma Miyoshi, Kensuke Tanioka, Shoko Yamamoto, Hiroshi Yadohisa, Tomoyuki Hiroyasu, Satoru Hiwa

**Affiliations:** ^1^Graduate School of Life and Medical Sciences, Doshisha University, Kyoto, Japan; ^2^Clinical Study Support Center, Wakayama Medical University Hospital, Wakayama, Japan; ^3^Department of Culture and Information Science, Doshisha University, Kyoto, Japan; ^4^Department of Biomedical Sciences and Informatics, Doshisha University, Kyoto, Japan

**Keywords:** mindfulness, breath-counting meditation, focused-attention meditation, fMRI, Tucker3 clustering, functional network, graph theoretical analysis

## Abstract

This study examines the effects of focused-attention meditation on functional brain states in novice meditators. There are a number of feature metrics for functional brain states, such as functional connectivity, graph theoretical metrics, and amplitude of low frequency fluctuation (ALFF). It is necessary to choose appropriate metrics and also to specify the region of interests (ROIs) from a number of brain regions. Here, we use a Tucker3 clustering method, which simultaneously selects the feature vectors (graph theoretical metrics and fractional ALFF) and the ROIs that can discriminate between resting and meditative states based on the characteristics of the given data. In this study, breath-counting meditation, one of the most popular forms of focused-attention meditation, was used and brain activities during resting and meditation states were measured by functional magnetic resonance imaging. The results indicated that the clustering coefficients of the eight brain regions, Frontal Inf Oper L, Occipital Inf R, ParaHippocampal R, Cerebellum 10 R, Cingulum Mid R, Cerebellum Crus1 L, Occipital Inf L, and Paracentral Lobule R increased through the meditation. Our study also provided the framework of data-driven brain functional analysis and confirmed its effectiveness on analyzing neural basis of focused-attention meditation.

## 1. Introduction

Mindfulness meditation is said to be influential in physicality, cognition, and mentality, and also has positive effects on well-being (Carmody and Baer, 2008; Chiesa and Serretti, 2009). Mindfulness-based stress reduction (MBSR) (Grossman et al., 2004) and mindfulness-based cognitive therapy (MBCT) (Teasdale et al., 2000) are clinical interventions based on mindfulness meditation, and it has been reported that these interventions alleviate symptoms of disorders, such as anxiety disorders (Roemer et al., 2008; Hofmann et al., 2010), depression (Teasdale et al., 2000), and substance use disorders (Bowen et al., 2006). Furthermore, it has also been reported that the practice of meditation contributes to improvement of well-being, quality of life (Carmody and Baer, 2008; Chiesa and Serretti, 2009), immune function (Davidson et al., 2003; Carlson et al., 2007), and cognitive function (Jha et al., 2007; Ortner et al., 2007; Pagnoni and Cekic, 2007; Slagter et al., 2007), not only for unhealthy people but also for healthy ones.

On the other hand, the biological mechanisms of mindfulness meditation have also been studied. In particular, the neural basis of mindfulness meditation has been investigated using noninvasive neuroimaging methods such as electroencephalography (EEG) (Davidson et al., 2003; Slagter et al., 2007) and functional magnetic resonance imaging (fMRI) (Farb et al., 2010; Goldin and Gross, 2010). For example, Short et al. have reported that expert meditators showed higher activation in the anterior cingulate cortex (ACC) and dorsolateral prefrontal cortex (dlPFC) during meditation (Baron Short et al., 2010), indicating that long-term practice of meditation affects brain function through effects on neuroplasticity. Hasenkamp et al. have revealed that there were four cognitive states during meditation: FOCUS (representing maintenance of attentional focus on the breath), MW (representing mind wandering or loss of focus), AWARE (representing the awareness of mind wandering), and SHIFT (representing shifting of focus back to the breath). They also found that the different brain regions were activated in each cognitive state (Hasenkamp et al., 2012). Their proposed model has been widely used to interpret the dynamics of the cognitive states during meditation. They have also reported that the functional connectivity between the dlPFC in the central executive network (CEN) and the right insula in the salience network (SN) was higher in experienced, long-term meditators compared with short-term meditators (Hasenkamp and Barsalou, 2012).

Most previous studies have focused on expert meditators and studied these two effects by comparing them with novices. Otherwise, most of the studies focusing on novice meditators have studied long-term effects by measuring brain activities of participants simultaneously with clinical intervention such as MBSR and MBCT (Chiesa and Serretti, 2009; Kilpatrick et al., 2011). However, difference in brain activity between resting- and meditative states in novice meditators has not been sufficiently studied. This is because meditating correctly is not easy for novices and it is believed that repeated practice for at least several weeks is necessary to achieve a proper meditative state.

Here, we investigate the differences in resting- and meditative states on brain function in novice meditators. Mindfulness meditation mainly includes focused attention (FA) meditation, which keeps attention to specific objects, and open monitoring (OM) meditation, which monitors current experience without value judgments. Practicing FA meditation is easier than OM meditation, and sustaining attention with an intention is one of the most important components of mindfulness, which is why FA meditation is the first method taught in many meditation training programs (Wallace, 2006; Lutz et al., 2008; Gunaratana, 2010). In addition, it has been reported that breath-counting meditation, which is a kind of FA meditation, improved psychological state and also reduced MW in novice meditators (Levinson et al., 2014). Therefore, FA meditation is one of the best methods for novices and is also used in this study.

In this study, the graph theoretical metrics of function networks are analyzed for novice meditators only, and the difference in network properties between resting and meditative states are compared each to investigate how the short-term practice of FA meditation affects the functional network. However, since there are a wide variety of graph theoretical metrics, such as degree centrality, betweenness centrality, and clustering coefficient, it is necessary to select the most suitable ones, such that the effects on the functional brain network are appropriately represented.

In addition, not only indicators showing relationships between brain regions, such as functional connectivity, but also those representing local activity, such as brain activation, are important for investigating brain function. Incidentally, in resting-state fMRI studies, the amplitude of low frequency fluctuations (ALFF) (Yu-Feng et al., 2007) that represents blood-oxygen-level-dependent (BOLD) signal power within the frequency band of interest (0.01–0.1 Hz) has been used as the indicator of local brain activity, and its correlation with the brain activation has also been reported in recent years (Kalcher et al., 2013). Thus, in this study, in addition to the graph theoretical indicators, fractional ALFF (fALFF), which is a modified version of ALFF, was used as the local activity indicator.

Although definition of brain region depends on the brain parcellation method, it is important but difficult to determine the region of interests (ROIs), because there are about 100 or more brain regions to be analyzed (e.g., 116 regions defined by automated anatomical labeling). Therefore, in this study, Tucker3 clustering (T3Clus) (Rocci and Vichi, 2005; Vichi et al., 2007), which simultaneously selects the feature vectors (graph theoretical metrics and fALFF) and the ROIs that best discriminate between resting and meditative states based on the characteristics of the given data, was used. T3Clus is a clustering method based on three-way principal component analysis using the Tucker3 model (Tucker, 1966) and classifies data while eliminating irrelevant features by reducing the dimension of data.

Here, we explain the outline of this study. First, the brain activities of the novice meditators were measured during a 5-min resting state and 5-min breath-counting meditation using functional magnetic resonance imaging (fMRI). Then, three graph theoretical metrics and fALFF, calculated for each brain region of all participants in both of resting and meditative states, were projected to 2D space by T3Clus, and the brain regions and feature indicators characterizing the difference between the two state were extracted. This difference was regarded as the effects of FA meditation in the novices, and its characteristics were investigated based on the selected feature vectors and ROIs.

## 2. Materials and Methods

### 2.1. Overview of the Proposed Method

Here we propose method to extract the meaningful feature vectors to distinguish between two experimental conditions, resting and meditative states. First, BOLD time courses during the two conditions were measured for all participants using fMRI. Second, three network feature vectors: degree centrality, betweenness centrality, and clustering coefficient (Bullmore and Sporns, 2009), and one local activity measure, fALFF, representing the intensity of spontaneous brain activity (Zou et al., 2008), were chosen and calculated to quantify the brain states. Finally, T3Clus was applied to classify the two experimental conditions, simultaneously decomposing original feature space into low dimensional space to maximize the classification accuracy. Here, we used the supervised T3Clus method, where the experimental conditions are used as the class label. The reason why T3Clus was chosen for the analysis was that it could deal with the mutual relationship among brain regions, network feature vectors, and local activity measure. It can consider all mutual dependencies between the different dimensions and provides a compact representation of the original tensor in lower- dimensional spaces (Fanaee-T et al., 2014).

### 2.2. Participants

Twenty-nine healthy adults (aged 22.9 ± 2.3 years, 6 females, all right-handed) participated in this experiment. Total hours of the meditation practice for each of them were less than 30 h, and none of them experienced daily meditation training. All participants were informed about the experimental method and the risk and signed written informed consents. This study was carried out in accordance with the research ethics committee of Doshisha University, Kyoto, Japan (approval code: 15098).

### 2.3. Data Acquisition

Whole-brain imaging data were acquired with a 1.5 T MR scanner (Echelon Vega, Hitachi, Ltd., Tokyo, Japan). Functional images were obtained using gradient echo-echo planer imaging (TR = 2,500 ms, TE = 40 ms, flip angle = 90°, FOV = 240 mm, 5.0-mm thick slices, matrix size = 64 × 64, number of slices = 25). We also employed an Rf-spoiled steady state gradient echo (RSSG) sequence to obtain T1-weighted structural images (TR = 9.8 ms, TE = 4.0 ms, flip angle = 8°, FOV = 256 mm, 1.0-mm thick slices, matrix size = 256 × 256, number of slices = 192).

### 2.4. Experiment

**Figure 1:**
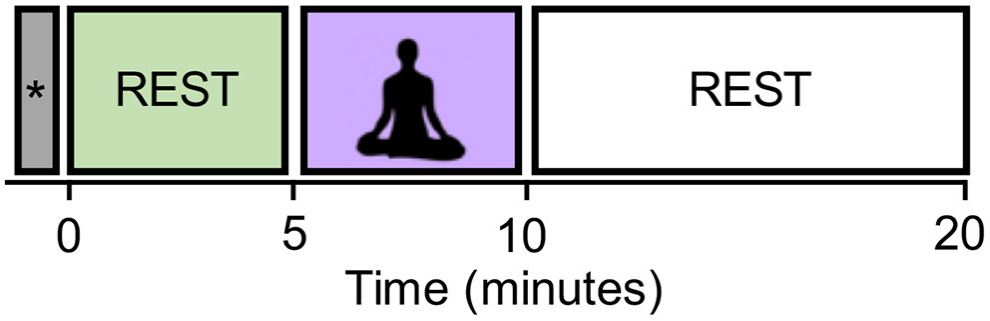
The experimental design. The block labeled with the symbol “*” contains the dummy six volumes (with the duration of 15 s) acquired before the start of the first resting block. They were excluded from the analysis in order to eliminate the non-equilibrium effects of magnetization.

The experiments consisted of a 5-min resting state block (pre-rest), a 5-min meditation block, and a 10-min resting state block (post-rest) as shown in Figure 1. Most of well-known mindfulness-baed interventions, such as MBSR, also have a minimum of 1–5 min of meditation practice (Carmody and Baer, 2008), and it is thought that sustained meditation practice at least for several minutes is necessary to achieve a stable meditative state. That is why we choose the 5-min meditation. Start and stop of meditation was informed by an auditory signal via headphones. The total duration of the experiment was 20 min. Participants practiced a simple-guided breath-counting meditation for a few minutes before entering the fMRI scanner. After the fMRI measurement, they were asked to rate (1) how correctly they could perform breath-counting meditation and (2) how frequently their attention wandered from breathing, on a scale of 1 (not at all) to 5 (did so successfully/wandered very frequently). If one rated 1 on the correctness of meditation, he/she was excluded from the analysis.

In the breath-counting meditation block, participants were asked to breathe through their nose and to try not to change the breath interval. They counted their breath silently from one to ten. They were also instructed to restart counting from one if they got to ten, or if their mind got distracted. Their eyes were kept closed consistently in the scanner. In the rest block, they were instructed to stay relaxed without focusing on their breathing. The dummy six volumes were acquired before the start of the first resting block and also excluded from the analysis in order to eliminate the non-equilibrium effects of magnetization.

We focused on the pre-rest and meditation blocks to see the differences in brain states induced by meditation. The post-rest block will be used to study another hypothesis, but was excluded for the current study.

### 2.5. Data Preprocessing

The fMRI data were preprocessed using SPM12 (Wellcome Department of imaging Neuroscience, London, UK) on MATLAB (MathWorks, Sherborn, MA). All functional images were corrected for motion effects using a six-parameter rigid body linear transformation and slice-time corrected to the middle slice. Then, T1-weighted anatomical images were coregistered to the mean of the corrected functional images. These functional images were normalized to the Montreal Neurological Institute/International Consortium for Brain Mapping (MNI/ICBM) standard and spatially smoothed using an isotropic Gaussian filter (FWHM = 8 mm). To minimize global drift effects, the signal intensities in each volume were divided by the mean signal value for the run and multiplied by 100 to produce percent signal change from the run mean. Participants showing head movements greater than 2 mm during each rest/task block were excluded from all subsequent analyses.

In addition to preprocessing through SPM12, the following, additional, preprocessing was performed. First, the whole brain was divided into gray matter, white matter, and cerebral spinal fluid (CSF). Then, nuisance regression was performed based on an anatomical component-based noise correction method (aCompCor) (Behzadi et al., 2007) to regress out the mean BOLD signals from the white matter and CSF and also remove head-movement and physiological confounding effects. Here, the main task effect from the meditation block (modeled as a canonical hemodynamic-response-function-convolved response) and its first order derivative were also regressed out to remove any potential confounding effects of shared task-related responses. Finally, a band-pass filter (0.008–0.09Hz) was applied to reduce the effects of physiological and low-frequency noise.

The preprocessed functional images were parcellated into 116 regions defined by automated anatomical labeling (AAL) and the mean BOLD time course was calculated for each region. These 116 time-courses were used to calculate the feature vectors.

### 2.6. Graph Theoretical Functional Network Analysis

Functional connectivity analysis is used to measure statistical interdependence (mutual information), without explicit reference to causal effects, by measuring correlation/covariance, spectral coherence, or phase-locking of time series brain activity between each brain region (Sporns et al., 2004). In this study, Pearson correlations were calculated between ROI-wise BOLD time courses during pre-resting and meditation blocks, and 116 × 116 functional connectivity matrices (FCMs) were obtained for each participant in each of the two conditions. The obtained FCMs were transformed to convert the sampling distribution of the Pearson correlation into a normal distribution. They were also binarized to determine the presence or absence of functional connections between the 116 regions. Determination of the appropriate thresholding method and its threshold value is one of the most crucial issues in graph theoretical functional network analysis. We used cost-based thresholding where the ratio of the connections with the strongest connectivity values out of the possible number of connections are preserved (e.g., cost 0.100 means the strongest 10% of possible of connections exist in the thresholded matrix). For threshold determination, we calculated the different threshold settings (from 0.050 to 0.500, increments: 0.025), and, then, the following three criteria were applied to them to choose the single threshold setting for the analysis.

*1) Small-world characteristics:* Based on the assumption that small world topology are seen in the functional networks (Achard et al., 2006; Honey and Sporns, 2008; Lynall et al., 2010), we calculated the global and local efficiency measures for all threshold settings, and then found that the cost range: 0.050–0.425 satisfied the small-world network criteria: higher global efficiency than a lattice graph but lower global efficiency than a random graph.

*2) Similarity to the average characteristics:* We aimed to determine the single threshold that can extract the network with group average characteristics between the different threshold settings (cost: 0.050–0.425). Therefore, we calculated the degree centrality measure for each brain region, for all participants, in each condition. Then, we averaged the degree centrality at different cost thresholds for each region within each participant, which were used as average characteristics of the degree centrality distribution. This processing was performed for each participant and each condition. To determine the single threshold that was the most similar to the average characteristics, for each participant, Pearson's correlation between the average degree distribution and the degree distribution of the FCM, thresholded by a certain cost value, were calculated for each cost setting. The correlation at each cost threshold was averaged between participants, and the cost value with the highest average correlation with the average degree distribution was chosen.

*3) Stability in the community structure:* If the network structure are stable across the cost, the number of communities existing in the thresholded network should be also stable (Brandl et al., 2017). Therefore, we used the number of communities detected by Newman clustering (Girvan and Newman, 2002) on the thresholded network as a cost criterion. The community clustering was performed for all the participants, all cost settings, in each condition. The standard deviation of the number of communities across participants was calculated for each cost. The cost setting with the smallest deviation was chosen for the stable cost threshold. Here, we set the parameter γ of Newman clustering to 1.0. If γ > 1, the smaller modules are detected while the larger modules are detected at 0 < γ < 1. We chose γ = 1 as a standard setting because we did not intend to bias the module size in stability analysis.

According to these three criteria, we got reasonable setting: cost = 0.225, and we reported results for the cost determination in Figure S1. Three graph theoretical metrics: degree centrality, betweenness centrality, and clustering coefficient were calculated as the indicators quantifying the network characteristics of each region (Bullmore and Sporns, 2009).

### 2.7. Fractional ALFF Calculation

We used fALFF measure to quantify the intensity of spontaneous brain activity induced or altered through meditation. ALFF is derived by calculating the sum of amplitudes of low-frequency of a specific frequency range, which is regarded as including spontaneous brain activity (i.e., 0.008–0.09 Hz was chosen for this study). It has been reported that the ALFF of the DMN is significantly higher in the resting state (Yu-Feng et al., 2007). The fALFF, used in this paper, is the modified form of ALFF obtained by dividing ALFF by the sum of the amplitudes of all the possible frequency bands to reduce the influence of noise (Zou et al., 2008). In this paper, fALFF were calculated for all voxels using CONN toolbox and then averaged within the brain region defined by AAL.

### 2.8. Tucker3 Clustering for Brain State Classification

T3Clus is a clustering method that simultaneously performs three-way principal component analysis and k-means clustering on the principal component scores (Tucker, 1966; Rocci and Vichi, 2005). Three-way data means that multiple objects were observed on multiple variables in multiple occasions. Here, a set of observation variables, X, are is organized as *I* × *J* × *K* three-way array, where *I*, *J*, and *K* denote the number of objects, variables, and occasions, respectively. With an increase of dimensionality of each of the three modes (objects, variables, occasions), the difficulty of the objects (data) classification will be increased (Milligan, 1996). In conventional approaches, one of the easiest ways to tackle this problem is to reduce the dimensionality by principal component analysis etc., and then perform the clustering. However, such an approach can result in loosing important prominent information which that contributes to classification through dimensionality reduction (Desarbo et al., 1991; De Soete and Carroll, 1994). In this case, T3Clus, in which the dimensionality of the variables and occasions are reduced so that the objects are the most classified by combining three-way principal component analysis and k-means clustering, is effective.

The Tucker3 model is represented by

(1) $$\begin{array}{l} {\text{X}}_{I,JK}=\text{U}{\text{Y}}_{G,QR}{\left(\text{C}⊗\text{B}\right)}^{\text{T}}+{\text{E}}_{I,JK} \end{array}$$

where **X**_*I,JK*_(*I* × *JK*), **Y**_*G,QR*_(G × QR), and **E**_*I,JK*_(*I* × *JK*) denote the “matricized” versions of the **X**(*I* × *J* × *K*), the centroid array **Y**(*G* × *Q* × *R*), and the residual array **E**(*I* × *J* × *K*), respectively. **U**(*I* × *G*), **B**(*J* × *Q*), and **C**(*K* × *R*) denote an indicator matrix defining a partition of the objects into *G* classes, a component loading matrix for variables, and ca component loading matrix for occasions, respectively, and ⊗ denotes the Kronecker product of matrices. Besides, *G*, *Q*, and *R* denote number of classes, components for variables, and components for occasions, respectively.

T3Clus solves the following optimization problem:

(2) $$\begin{array}{l} \text{Minimize }F_{\text{T}3\text{C}}\left(\text{B},\text{C},\text{U},\text{Y}\right)=||{\text{X}}_{I,JK}-\text{U}{\text{Y}}_{G,QR}{\left(\text{C}⊗\text{B}\right)}^{\text{T}}{||}^{2} \end{array}$$

subject to **B** and **C** column-wise orthonormal and **U** binary and row- stochastic.

In this study, functional neuroimaging data were treated as three-way data consisting of 29 participants, 116 brain regions, and four regional functional metrics, degree centrality, betweenness centrality, clustering coefficient, and fALFF. Here, generally, indicator matrix U is constrained to have only one nonzero element per row to indicate which class each row belongs to, and it is optimized for solving the above optimization problem. However, in this study, we determined each element of U without the optimization process because it was obvious which experimental conditions (i.e., resting or meditative states) each data belonged to.

It should be noted that T3Clus leads to derivation of linear transformation from the original data space into low-dimensional space by optimizing the Kronecker product of *C* and *B*. Therefore, the component scores after executing T3Clus can be obtained by:

(3) $$\begin{array}{l} {\text{Y}}_{I,QR}=\text{U}{\left({\text{U}}^{\text{T}}\text{U}\right)}^{-1}{\text{U}}^{\text{T}}{\text{X}}_{I,JK}\left(\text{C}⊗\text{B}\right) \end{array}$$

**Figure 2:**
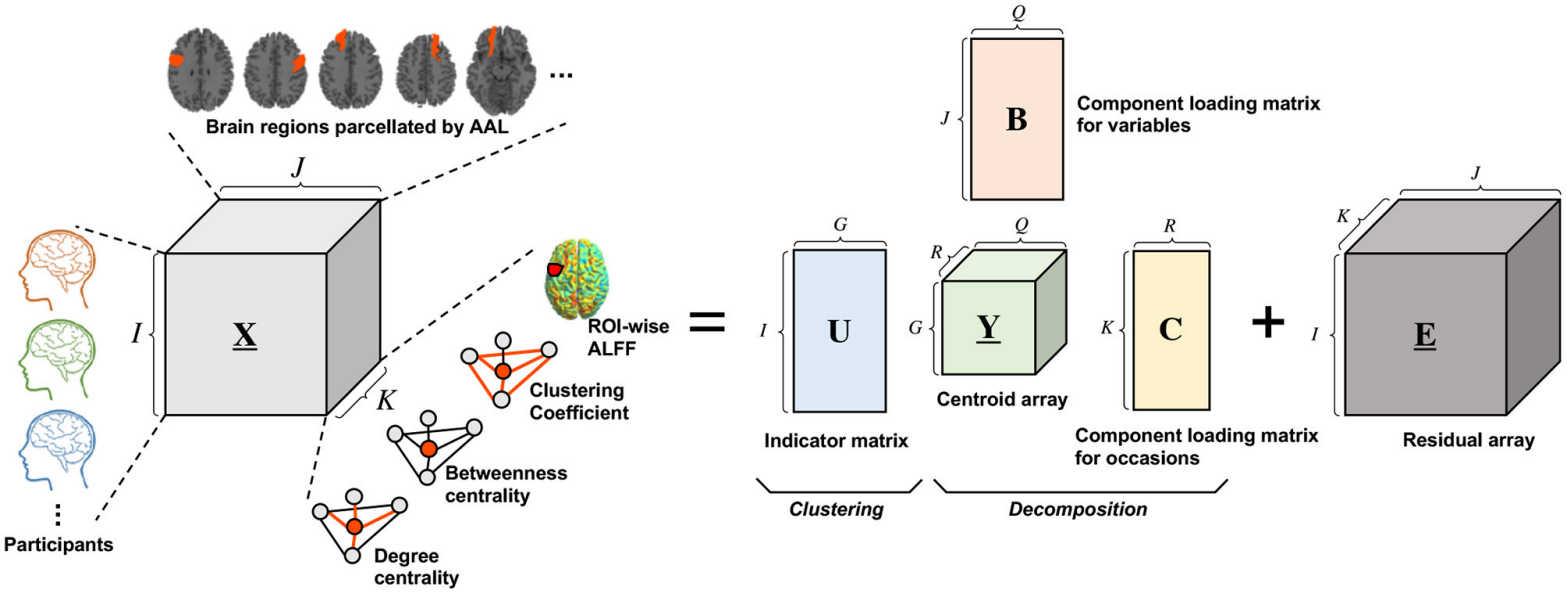
Schematic illustration of Tucker3 clustering on functional neuroimaging data. X(*I* × *J* × *K*), Y(*G* × *Q* × *R*) and E(*I* × *J* × *K*) denote the three-way data array of observation data, the centroid array, and the residual array, respectively. U(*I* × *G*), B(*J* × *Q*), and C(*K* × *R*) denote the indicator matrix defining a partition of the objects into *G* classes, the component loading matrix for variables, and the component loading matrix for occasions, respectively.

The schematic illustration of T3Clus on functional neuroimaging data is indicated in Figure 2. In this study, the 29 participants' data were classified into two classes, simultaneously with reducing the number of brain regions from 116 to 2 and the number of functional metrics from 4 to 1 dimension using T3Clus (i.e., *G* = 2, *Q* = 2, and *R* = 1). As a result, the two-dimensional component scores of the neuroimaging data during the resting and meditative states of the 29 participants were obtained and were plotted in two-dimensional space.

### 2.9. Finding Discriminating Brain Regions and Their Features

Since the component scores are obtained by Equation (3), the Kronecker product **C** ⊗ **B** can be regarded as a weight vector to the observation data. Thus, its elemental value can also be treated as the metric indicating the importance of each row or column of the three-way array. In the meditation dataset, the higher elemental value indicates that the corresponding feature is important for to discriminating between the brain states between under resting- and meditation conditions.

Here we aim to elucidate the essential combination of the brain regions and their functional brain metrics by analyzing the component loading matrix **C** ⊗ **B**. That is, discriminating features are extracted from the elements of the component- loading matrix whose values are necessary to discriminate between resting- and meditation conditions.

In order to achieve this, we applied the permutation tests (Bullmore et al., 1996; Holmes et al., 1996; Tegeler et al., 1999; Nichols and Holmes, 2002; Mourao-Miranda et al., 2005) to the component- loading matrix. The class labels of the state were randomly permuted 10,000 times and T3Clus was applied to the data set of each permuted label. The probability that loading with a larger absolute value than the loading obtained from original data set was given was the *p*-value. The significant brain regions and feature values were extracted under *p*-values < 0.05.

### 2.10. Performance Comparison With the Conventional Approach

To ensure the effectiveness of applying T3Clus to our study in terms of finding the prominent features to discriminate different cognitive conditions, the paired *t*-test was applied between resting and meditative state in each of four feature values, and then the brain regions whose feature values were significantly different between two conditions were extracted (*p* < 0.05, FDR-corrected) and compared with the results obtained by T3Clus.

### 2.11. Verification of Dependence of the Computational Results on the Network Threshold

We described how to choose the single network threshold to extract the representative network structure in a given experimental condition, in section 2.6. However, the computational results would vary depending on the choice of network threshold. Here, we investigate whether the results of the specific threshold stable or not if a different threshold was chosen. To examine this, T3Clus was performed at different threshold settings (from 0.050 to 0.500, increments: 0.025), and then the obtained results were compared between the different thresholds.

## 3. Results

### 3.1. Subjective Ratings

Average rating of the correctness of meditation was 3.6 ± 1.1 (1: not at all, 5: did so successfully) and nobody rated 1 so that all the participants' data were used for the following analysis. Besides, average rating of the mind wandering was 3.2 ± 0.7 (1: not at all, 5: very frequently).

### 3.2. Paired *t*-Test

The paired *t*-test performed on each of the four feature metrics for each brain region between resting and meditative states revealed that there were no significant differences between two states in all brain regions for all feature values (*p* > 0.05).

### 3.3. Low-Dimensional Representation of the Brain States Derived by T3Clus

**Figure 3:**
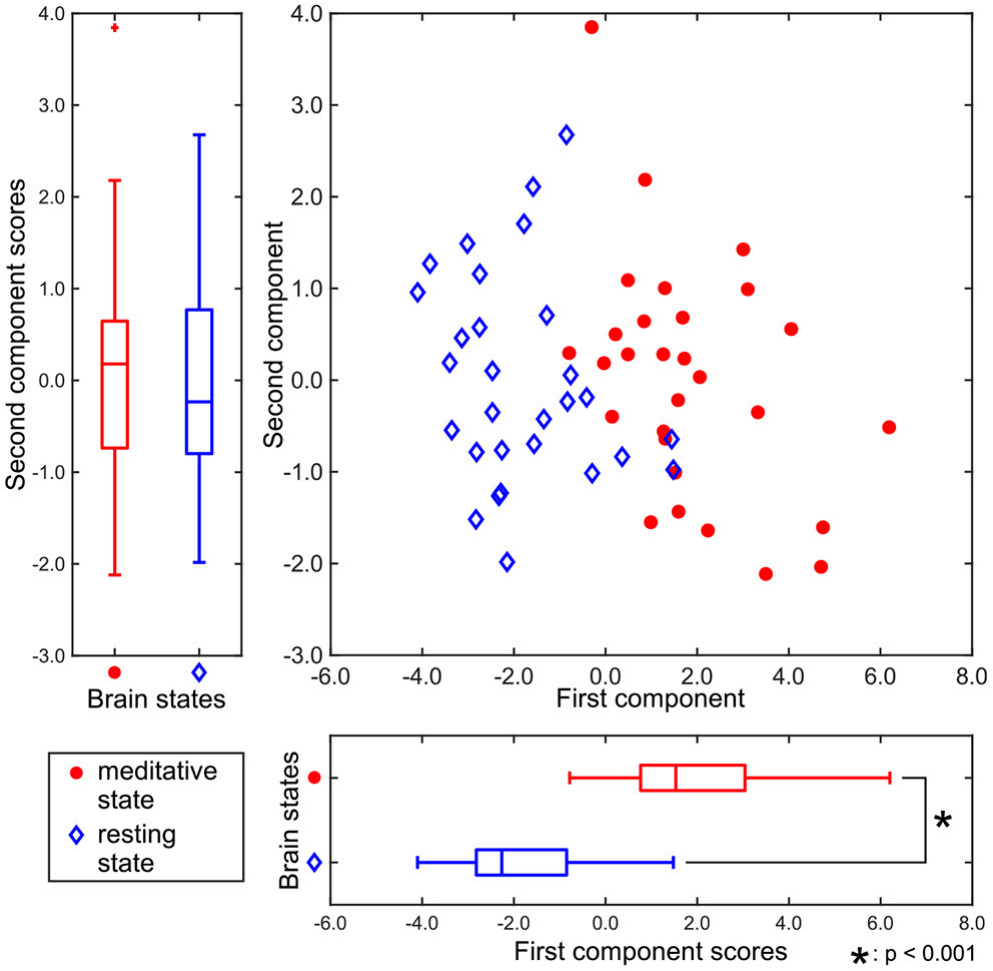
Component scores obtained by T3Clus. Each point indicates each participant's data. The meditative state is red-colored and the resting state is colored blue.

Figure 3 shows the two-dimensional representation of each participant's brain state during the two experimental conditions, obtained by T3Clus. Each axis was determined by T3Clus to maximize the difference between two conditions, simultaneously reducing the dimensionality of the brain regions and feature metrics.

**Figure 4:**
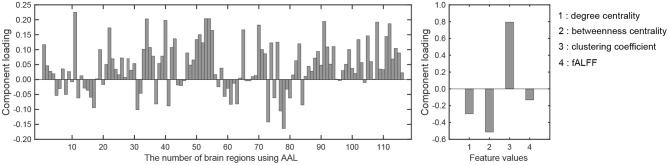
Component loadings obtained by T3Clus. **(Left)** Loadings for brain regions. **(Right)** Loadings for four feature values.

There was a significant difference between the first component scores in the resting and meditative conditions (*p* < 0.001), while there was a no significant difference between the second component scores. Therefore it can be said that the discriminating brain regions and features to classify between two states exist in the component loadings for the first component. The component loadings for the first component are shown in Figure 4.

To extract the essential brain regions and their feature values, the permutation test was performed on the component loading matrix. Tables 1, 2 indicate the significant brain regions and feature values with *p*-values < 0.05, respectively. The significant brain regions shown in Table 1 were the top 8 largest component-loading values in Figure 4. On the other hand, for the feature values, only the clustering coefficient was extracted as a significant discriminating feature.

**Table 1:** First component loading of brain regions (*p* < 0.05).

**Brain region**	**Loading**	**p-score**
Frontal Inf Oper L	0.2250	0.0125
Occipital Inf R	0.2036	0.0215
ParaHippocampal R	0.1981	0.0224
Cerebellum 10 R	0.1923	0.0281
Cingulum Mid R	0.2031	0.0284
Cerebellum Crus1 L	0.1946	0.0309
Occipital Inf L	0.2036	0.0324
Paracentral Lobule R	0.1824	0.0397

**Table 2:** First component loading of feature values (*p* < 0.05).

**Feature value**	**Loading**	**p-score**
Clustering coefficient	0.7943	0.0370

**Figure 5:**
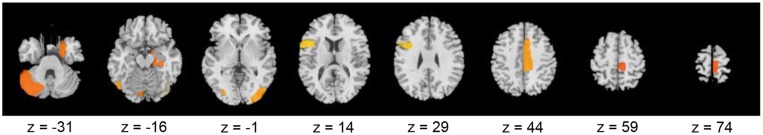
Component loading maps of the eight brain regions that contribute to the classification between resting- and meditative states.

Therefore, these results indicated that the eight brain regions and their clustering coefficients were essential for discriminating brain states between resting and meditative conditions. Figure 5 shows the component loading maps of the eight brain regions that contribute to the classification of the two states, displayed on axial slices at different *z* levels.

### 3.4. Dependence of the Computational Results on the Network Threshold

**Figure 6:**
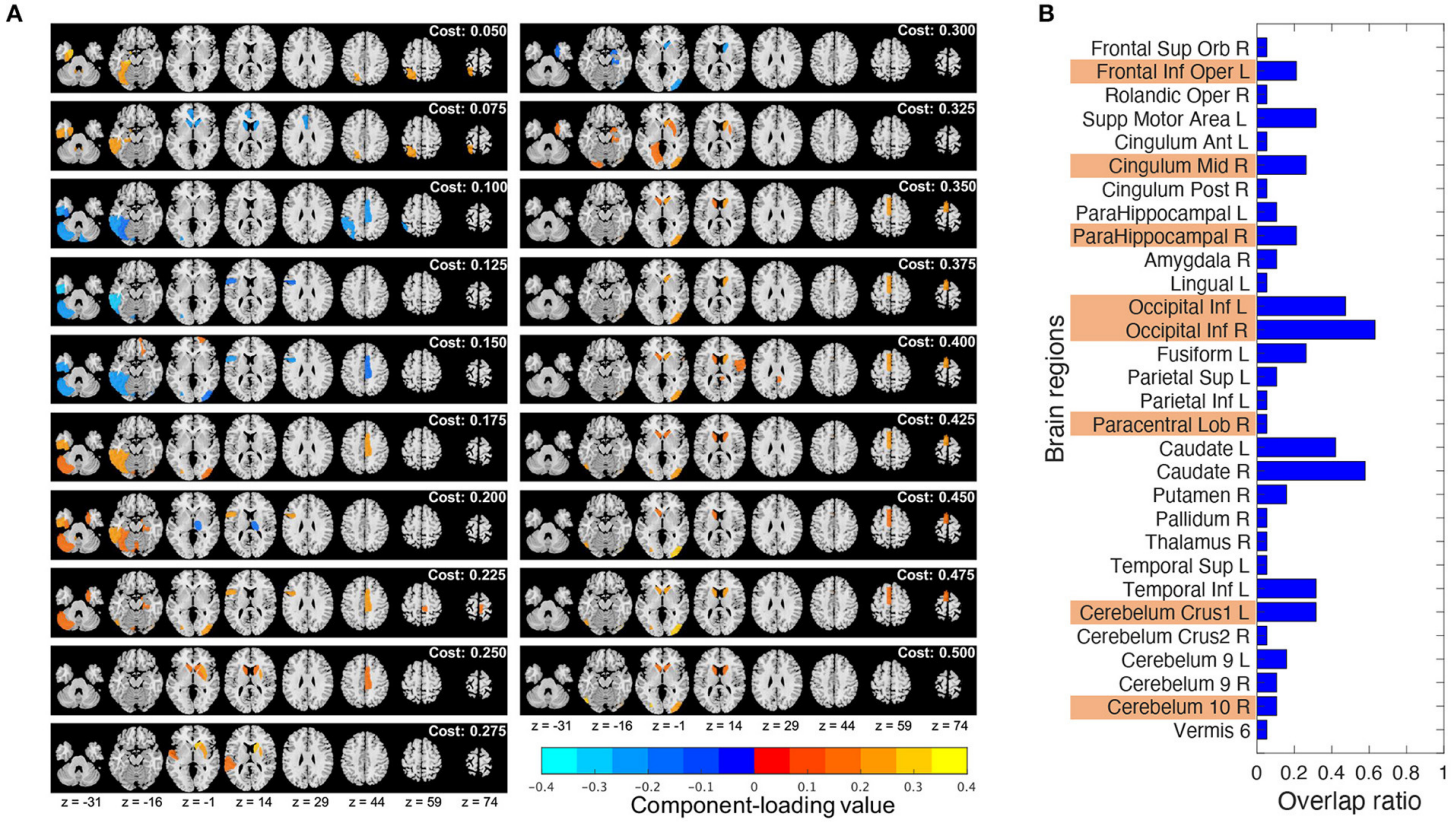
Comparison of the T3Clus results between different network thresholds (from 0.050 to 0.500, increments: 0.025). **(A)** Component loading maps at different thresholds (referred to as “cost” in the figure). **(B)** Overlap ratio of the chosen brain regions among 19 different cost values. Orange colored ones are the eight regions extracted at cost = 0.225.

Component loading maps of the different network thresholds (costs) were summarized in Figure 6A. Additionally, to check how much stable the chosen brain regions were, the ratio of the brain regions chosen by T3Clus which were overlapped among the different costs to the total number of cost settings (19) was calculated and shown in Figure 6B. The brain states whose overlap ratios exceeded 0.40 were Occipital Inf L/R and the left and right caudate (Caudate L/R), and their values were 0.47 (L), 0.63 (R), 0.42 (L), and 0.58 (R), respectively.

## 4. Discussion

### 4.1. Performance Comparison Between T3Clus and the Conventional Approach

According to the conventional approach, *t*-test results revealed that there were no brain regions that significantly discriminated between resting and meditative states for all four feature values. This result suggests that it is difficult to explain the functional brain changes caused by meditation with a single functional brain metric.

On the other hand, when feature space was constructed considering the dependency between multiple variables by T3Clus, two states were classifiable and the discriminating features could be extracted. It is suggested that T3Clus can extract meaningful features that potentially affect classification between the two states. Additionally, these results also suggest that the differences between the resting- and meditative states in novice meditators are observed only in the projected space constructed by T3Clus.

### 4.2. Differences Between Resting- and Meditative States Induced by Breath-Counting Meditation

In Table 2, only the clustering coefficient was extracted as the discriminating feature. It quantifies the number of connections that exist between the nearest neighbors of a certain node as a proportion of the maximum number of possible connections (Watts and Strogatz, 1998). With an increase in the clustering coefficient, the node tends to form with a high density of connections. In addition, it is notable that fALFF was not extracted. This suggests that local activity is not altered by performing the breath-counting meditation, while the functional network structure is affected.

In Tables 1, 2, all the component loadings of the eight brain regions and the feature value (clustering coefficient) constructing the first component were positive. Component scores can be calculated by the product of the observation values and the weights, which are obtained by the product of the loadings of the brain regions and the loading of the feature value. Therefore it indicates that the clustering coefficients of the eight brain regions are positively correlated with the first component score. Since the first component could discriminate between resting and meditative states, and the meditative states were distributed on the positive side of the first component in Figure 3, the clustering coefficients of the eight brain regions in the meditative state were higher than those in the resting state.

Next, we give an interpretation to each of the eight brain regions in Table 2. The right parahippocampal gyrus (ParaHippocampal R) located at the limbic system was extracted as one of the discriminating regions and plays an important role in creation of memories and recall of visual scenes (Aminoff et al., 2013). It is also known as a part of the DMN (Fransson, 2006). Furthermore, it has been reported that the left crus I of the cerebellar hemisphere (Cerebellum Crus1 L) is a part of the DMN (Favaro et al., 2012; Lavagnino et al., 2014). The inferior occipital cortex (Occipital Inf) is included in the visual regions of the occipital region (Beckmann et al., 2005; Damoiseaux et al., 2006; Veer et al., 2010) and also is a part of the DMN (Fox et al., 2005; Fransson, 2005). It has been reported that the DMN is activated in MW states, where the participant's attention is distracted from breathing (Hasenkamp et al., 2012). Our results suggested that the DMN regions, the ParaHippocampal R, Cerebellum Crus1 L, and Occipital Inf formed dense connections with other regions in moving from the resting state to the meditative state.

The right middle cingulate gyrus (Cingulum Mid R) is included in a fronto-parietal network (FPN), which is involved in top-down attention and controlling task execution (Corbetta et al., 2008; Craig and Craig, 2009). The opercular part of left inferior frontal gyrus (Frontal Inf Oper L) is also included in the FPN. It has been shown that the FPN is formed when the meditators sustain their attention on their breathing appropriately (Hasenkamp et al., 2012). Therefore, it is expected that the densities of the networks that include the FPN regions, Cingulum Mid R, and Frontal Inf Oper L as nodes would be increased by sustaining the attention on breathing during meditation.

In addition, the right paracentral lobule (Paracentral Lobule R) is part of the somatosensory network (SSN) (Favaro et al., 2012; Lavagnino et al., 2014), and it has been reported that this network is activated when the meditator's attention shifts back to the breath after being aware of MW (Hasenkamp et al., 2012). This suggests that the Paracentral Lobule R is densely connected with other regions because the participants noticed MW in the meditation block and tried to return their attention to breathing again.

Each of three networks, DMN, FPN, and SSN, are known as characteristic functional network associated with the cognitive cycle that occurs during meditation. Based on our findings, we hypothesize that the changes in the brain states induced by the breath-counting meditation is that each of the eight brain regions, mainly those included in the three networks, form a densely-connected network.

### 4.3. Effects of the Network Threshold Choice on the Computational Results

From Figure 6B, in the choice of the single cost value of 0.225, the most stable brain regions among the different cost values were Occipital Inf L/R. The other regions were dependent on the choice of cost values.

Notably, Caudate L/R which were not chosen as the discriminating regions at cost = 0.225 were highly overlapped among the different cost values. However, from Figure 6A, we can observe that Caudate L/R tended to be chosen at higher cost values. Actually, if we calculated the overlap ratios of Caudate L/R in each of lower (from 0.050 to 0.225) and higher (from 0.250 to 0.500) cost ranges, those of the lower range were 0.13 (L) and 0.13 (R), and those of the higher range were 0.64 (L) and 0.91 (R). Gard et al. (Gard et al., 2015) revealed that yoga practitioners and meditators had significantly greater degree centrality in the caudate than controls, and also suggested that meditators would have stronger functional connectivity within basal ganglia cortico-thalamic feedback loops than non-practitioners.

From these results, it can be expected that different cost choice would provide different characteristic brain regions and network structure. However, we need to be careful that the higher the cost values are, the more the network connections with lower functional connectivity are taken into account for representation of the brain states. This would lead to overemphasize weak connections. In general, the network thresholding are applied to reduce the computational burden of analyzing the network by removing weak connections (Garrison et al., 2015). Therefore we believe it is necessary to choose the specific cost value that balances between the computational burden and extracting meaningful network structure based on the some sort of criterion.

### 4.4. Limitations of the Current Study and Future Directions

In this study, we gave some interpretations of the brain regions and network features chosen by T3Clus. For example, several regions in DMN were observed in our results, however, the classical regions such as posterior cingulate cortex, precuneus, medial prefrontal cortex and inferior parietal were not involved in the current results. To make our interpretations more reliable, the hypothesis-driven study where the brain regions chosen by T3Clus are set to the region-of-interests and also their functional network characteristics are used for hypothesization should be further performed after the T3Clus execution. Another future direction is to review the experimental design to measure the behavioral metrics quantifying the quality of meditation. This would lead to make our interpretations more reliable.

Nonetheless, the major contributions of our study are that (1) we provided the framework of data-driven approach using T3Clus on graph theoretical metrics and spontaneous local activity measure, and (2) also indicated the differences between resting- and meditative states (during FA meditation) in novice meditators: the eight brain regions, Frontal Inf Oper L, Occipital Inf R, ParaHippocampal R, Cerebellum 10 R, Cingulum Mid R, Cerebellum Crus1 L, Occipital Inf L, and Paracentral Lobule R are densely connected with other regions through FA meditation. We believe these results are meaningful because they can't be derived by ordinary independent variable analysis.

## 5. Conclusion

In this study, we investigated the differences between resting- and meditative brain states induced by focused-attention meditation in novice meditators. In the experiment, breath-counting meditation, one of the most popular forms of focused-attention meditation, was used, and brain activity during resting and meditation states was measured by fMRI. Functional changes in brain states were analyzed by the T3Clus method applied to the three graph theoretical metrics, degree centrality, betweenness centrality, and clustering coefficient and one spontaneous local activity measure, fALFF, calculated from the fMR images measured.

The results indicated that the two experimental conditions could be differentiated based on the first component of the two-dimensional feature space identified by T3Clus. Moreover, the component loadings of the first component revealed that the clustering coefficients of the eight regions were the prominent features to discriminate between resting and meditative states. We also found that the clustering coefficients of these regions tended to be higher in the meditation state. The extracted regions were included in either of three networks, DMN, FPN, and SSN, which are known to be characteristic functional network associated with the cognitive cycle that occurred during meditation. Our results revealed that the changes in the brain activity induced by breath-counting meditation can be explained by the network density changes in these eight brain regions. It should be noted that no significant differences have been found, by *t*-test, in each of the four feature metrics for each brain region between two experimental conditions. This suggests that only T3Clus could detect the differences between resting- and meditative brain states by removing the feature metrics and the brain regions irrelevant to the functional changes caused by meditation.

## Data Availability Statement

The datasets generated for this study are available on request to the corresponding author.

## Ethics Statement

The studies involving human participants were reviewed and approved by Research ethics committee of Doshisha University, Kyoto, Japan. The patients/participants provided their written informed consent to participate in this study.

## Author Contributions

TM, TH, and SH designed the study and wrote the manuscript. TM, KT, SY, and SH conducted data analysis. HY and TH advised on the proposed method. All authors reviewed the manuscript.

### Conflict of Interest

The authors declare that the research was conducted in the absence of any commercial or financial relationships that could be construed as a potential conflict of interest.
