# Supplementary material for: Revealing Changes in Brain Functional Networks Caused by Focused-Attention Meditation Using Tucker3 Clustering
Source: Front Hum Neurosci. 2020 Jan 22;13:473. doi: 10.3389/fnhum.2019.00473 (PMC6990115; doi:10.3389/fnhum.2019.00473)
Supplement: Supplementary file 1 [file Data_Sheet_1.PDF]

Supplementary Material:
Revealing changes in brain functional networks caused by focused-attention meditation using Tucker3 clustering

a)

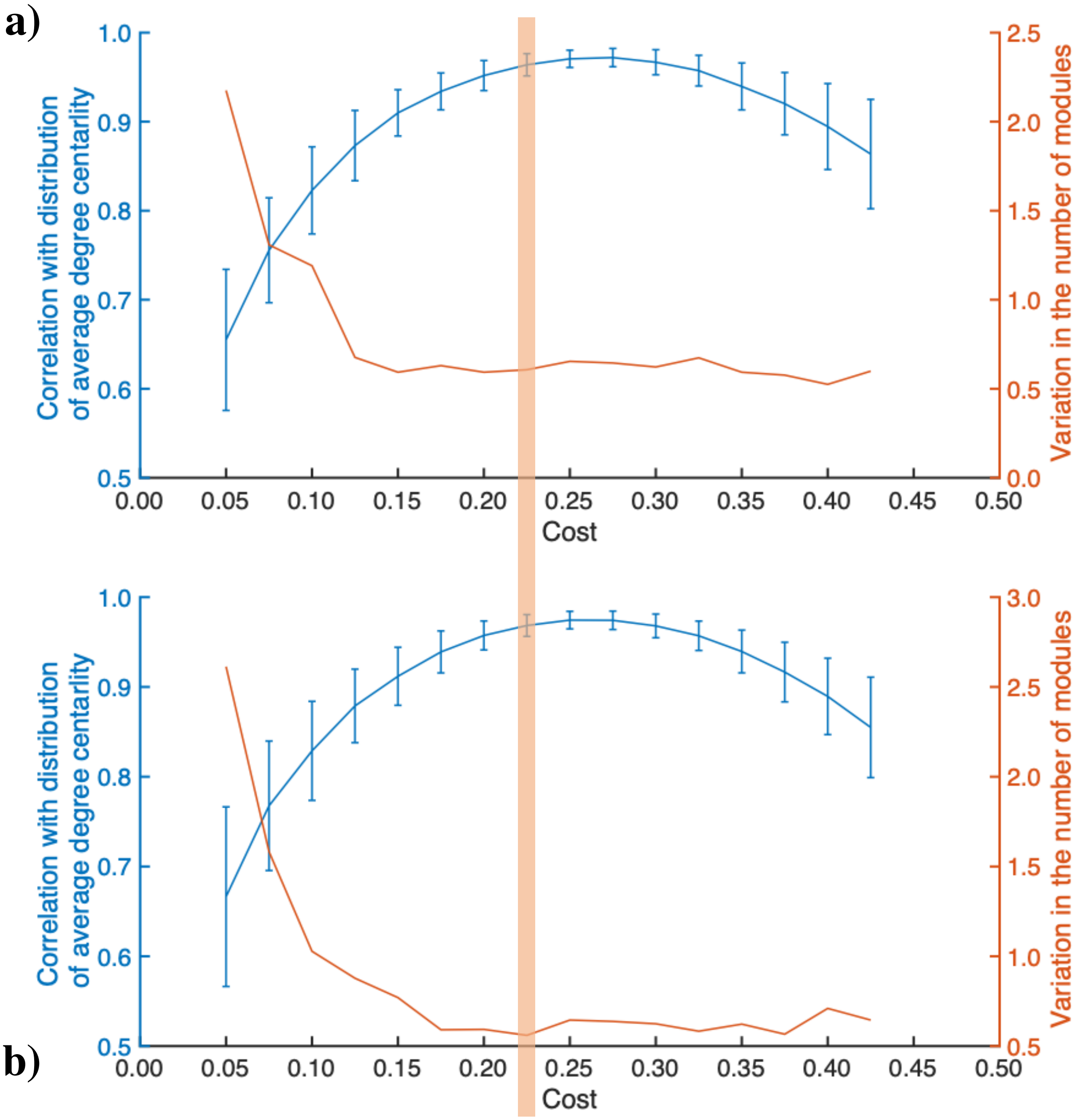

b)

Figure S1. Across-individual average correlation between the average degree distribution and the degree distribution of the FCM, thresholded by the different threshold settings (from 0.050 to 0.500, increments: 0.025) is plotted in the left axis. The standard deviation of the number of communities across participants, calculated for each cost, is also plotted in the right axis. The distributions of two metrics were calculated for a) resting state and b) meditative state.
